# Escalating pyrethroid resistance in *Anopheles funestus* increases *Plasmodium* transmission

**DOI:** 10.1186/s12936-026-06094-6

**Published:** 2026-08-13

**Authors:** Brice Natchema Soh Fonkou, Magellan Tchouakui, Benjamin Menze, Jeannette Tombi, Charles Sinclair Wondji

**Affiliations:** 1 Medical Entomology Department, Centre for Research in Infectious Diseases (CRID), Yaoundé, Cameroon; 2 Faculty of Sciences, University of Yaoundé I, 337, Yaoundé, Cameroon; 3 Vector Biology Department, Liverpool School of Tropical Medicine, Pembroke Place, Liverpool, L3 5QA UK

**Keywords:** *An. funestus*, Resistance escalation, Malaria transmission, Vectorial capacity, *GSTe2.*

## Abstract

**Background:**

The swift rise in pyrethroid resistance among malaria vectors poses a significant challenge to global malaria control. Although vector control tools have advanced, limited understanding of the vectorial capacity of highly pyrethroid-resistant mosquitoes hinders accurate predictions of how new interventions will affect malaria transmission in these resilient populations. Consequently, further research is needed to elucidate how escalating resistance influences mosquito vectorial capacity and shapes malaria transmission dynamics.

**Methods:**

Wild *Anopheles funestus* s.l. mosquitoes were sampled from households in Adamaoua and Centre regions of Cameroon between February and September 2021 using a Prokopack aspirator. Phenotypic resistance to pyrethroids (permethrin, deltamethrin, alphacypermethrin) was assessed using WHO tube bioassays at diagnostic dose (DD), as well as at 5 × and 10 × DD. *Plasmodium* sporozoite infections were detected using TaqMan and nested PCR assays, and key resistance markers were genotyped by allele-specific PCR. Pearson’s chi-squared tests and odds ratio were applied to examine the associations between resistance phenotypes, genotypes, and infection status.

**Results:**

Mosquitoes from Cameroon displayed low mortality rates in Mibellon, with only 33% mortality at the diagnostic dose (DD) and less than 95% mortality at 5 × and 10 × DD. In contrast, Elende populations exhibited lower resistance intensity. Sporozoite infection rates were very high in both collection sites (8.8% and 6% in Mibellon and Elende, respectively). Mosquitoes killed by elevated doses of type II pyrethroids were significantly more infected with *Plasmodium* than survivors (*p* < 0.05). Additionally, those surviving the highest deltamethrin doses showed reduced vectorial capacity compared to those surviving the 1 × diagnostic dose (OR = 2.8; 95% CI 1.1–7.3; *p* = 0.041). The persistence of elevated *Plasmodium* infection rates in mosquitoes surviving the highest permethrin concentrations underscores the direct impact of pyrethroid resistance escalation in driving malaria transmission. Genotypic analysis identified the L119F-*GSTe2* mutation as associated with both resistance escalation and increased *Plasmodium* transmission whereas an opposite effect was observed for the P450 *CYP9K1* with homozygote resistant 454A-*CYP9K1* less infected than susceptible.

**Conclusions:**

These findings highlight the urgent need for effective resistance management strategies to combat escalating pyrethroid resistance, while supporting the sustained use of type II pyrethroids, which remain effective against key *Plasmodium*-transmitting populations.

**Supplementary Information:**

The online version contains supplementary material available at https://doi.org/10.1186/s12936-026-06094-6.

## Background

Malaria vector control heavily relies on the use of long-lasting insecticidal nets (LLINs) and indoor residual spraying (IRS), which played a pivotal role in reducing malaria incidence and mortality across Africa between 2000 and 2015 [1–4]. However, since 2015, neither incidence nor mortality have reduced substantially as evidenced by the recent World Health Organization (WHO) malaria report, which estimates 282 million cases and 610 000 deaths in 2024 [3, 4]. The World Health Organization’s High Burden to High Impact (HBHI) initiative prioritizes a small number of countries that collectively contribute roughly two-thirds of the global malaria burden, representing approximately 67% of malaria cases and 73% of deaths globally in 2022 [4]. Cameroon, as one of these HBHI countries, contributes to this disproportionate share of morbidity and mortality, consistent with the continued high burden observed across the WHO African Region [1, 4, 5]. Malaria transmission in the country is favored by a highly diverse anopheline fauna exhibiting contrasting behaviors and physiological responses to interventions [6, 7]. Among these, *Anopheles funestus* s.l. has established itself as an exceptionally dominant vector with a very high annual entomological inoculation rate (EIR = 792 infected bites received per person per year), as recently reported in northern and southern Cameroon, where it is alone responsible for 98.6% of all annual transmission events [7].

In *An. funestus* s.l., as in other major malaria vectors, studies have documented high levels of resistance to all four classes of insecticides recommended by the WHO across Cameroon [8–10]. Resistance to pyrethroids particularly, the primary insecticide class used in bed nets and indoor residual spraying (IRS), has escalated dramatically, largely due to their extensive use in both agricultural practices and vector control programs [8, 9, 11]. This is a major concern because it can lead to a loss of efficacy of insecticide-treated nets (LLINs), including those containing piperonyl butoxide (PBO) [2, 11–13]. In this species, resistance escalation was associated with a massive overexpression of cytochrome P450 genes and other metabolic genes such as glutathione S-transferase (GST) genes and carboxylesterases (COE) combined with behavioral and cuticular resistance mechanisms [9, 11, 12, 14–16]. To date, key genetic resistance markers detected include the L119F*-GSTe2* mutation, associated with DDT/pyrethroids [17, 18]; the duplicated *CYP6P9a/b* associated with carbamate, neonicotinoid and pyrethroid cross-resistance [15] and negatively associated with chlorfenapyr resistance [19]; and the *CYP6P4a/b mutations (*M220I-*CYP6P4a* and D284E*-CYP6P4b)* [20], G454A*-CYP9K1* [21] and the overexpression of the *CYP325A* [22], all linked to pyrethroid resistance.

Insecticide exposure and the subsequent development of resistance in anopheline mosquitoes are critical factors influencing *Plasmodium* transmission. However, the precise effects of insecticide resistance on mosquito vectorial capacity and competence are complex and not fully understood. Despite extensive characterization of insecticide resistance and the identification of associated molecular markers, the real-world impact of this resistance on malaria infection and transmission remains poorly defined, necessitating further research to elucidate these crucial relationships.

Insecticide exposure can significantly alter the vectorial capacity of infected mosquitoes. In fact, pyrethroid exposure often increases mortality rates, reducing the number of mosquitoes capable of transmitting sporozoites and thereby decreasing overall vectorial capacity [23–25]. Moreover, *Plasmodium* infection may make mosquitoes more vulnerable to insecticides by impairing their immune systems and physiological conditions [26]. In addition to affecting mosquito lifespan, insecticide exposure can directly impact *Plasmodium* development, even in the absence of known molecular targets within the parasite. Research indicates that resistant anopheline populations show decreased parasite prevalence or intensity after sublethal exposure to various insecticides, including bendiocarb, DDT [27], permethrin [28], deltamethrin [26], and chlorfenapyr [29, 30]. Conversely, surviving mosquitoes after exposure may undergo biological adaptations, demonstrating behavioral changes such as increased host-seeking activity during dawn, early morning, or even in daylight [31–33]. These adaptations can enhance their vectorial capacity despite the initial mortality effects of insecticide exposure. Previous genetic studies have shown that *Anopheles* mosquitoes resistant to insecticides, particularly *An. funestus s.s*. carrying the L119F-*GSTe2* mutation, often maintain or even exhibit enhanced vectorial capacity. This mutation has been linked to heightened *Plasmodium falciparum* oocyst intensity [34], sporozoite prevalence [35] and entomological inoculation rates [7]. Similarly, target-site mutations like *kdr* and *Ace*-1 in *An. gambiae* have been linked to higher *P. falciparum* prevalence and parasite load [36–39]. However, other studies indicate that insecticide resistance may impair [35, 36, 38] or have neutral effects [7, 40, 41] on vectorial capacity, underscoring the complex interaction between insecticide resistance mechanisms and plasmodium infection/transmission and thus mosquitoes’ vectorial capacity.

While new tools such as chlorfenapyr-pyrethroid LLINs and pyrethroid-PBO nets offer some promise in combating pyrethroid resistance and enhancing protection from malaria [25, 42, 43], their effectiveness depends on a comprehensive understanding of the interplay between resistance, parasite infection, and mosquito vectorial capacity. The vectorial capacity of highly pyrethroid-resistant mosquitoes that survive elevated insecticide exposures (5 × and 10 × diagnostic doses) remains insufficiently characterized, hindering our ability to predict how novel vector control tools will influence malaria transmission sustained by these resilient mosquito populations. Therefore, more investigation on the impact of resistance escalation on the vectorial capacity of mosquitoes is needed. In this study, having determined the intensity of pyrethroid resistance in wild-caught *An. funestus* (F0) mosquitoes with exposure at 1X, 5X and 10X, we evaluated the impact of resistance escalation on their vectorial capacity revealing an increased contribution of highly resistant mosquitoes to malaria transmission.

## Methods

### Study sites

**Fig. 1:**
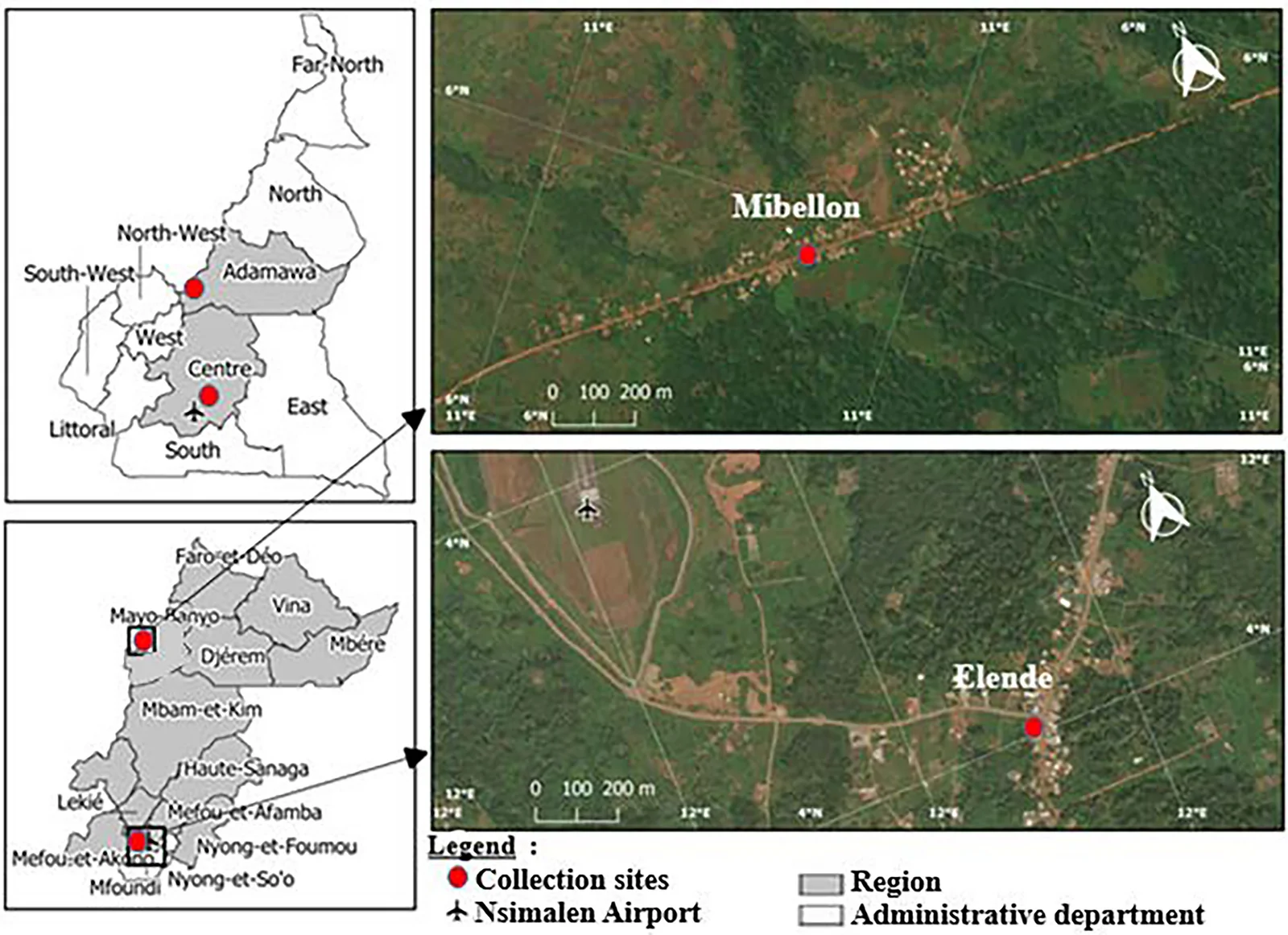
Mibellon and Elende study sites

The study was conducted in two different eco-epidemiological sites in Cameroon: Elende (3° 41′ 57.27″ N, 11° 33′ 28.46″ E) in the equatorial rainforest and Mibellon (6° 4′ 60 N, 11° 30′ 0E) in the humid savannah [7] (Fig. 1).

Mibellon is a village located in Central-northern Cameroon (Adamaoua region) at 350 km from Yaoundé (the country’s capital city). The climate is Sudano-Guinean, characterised by a five-month rainy season from March to April and from August to October, and a seven-month dry season extending from November to February and from May to July [44]. The village is located close to permanent water bodies, including a lake and swamps, which provide suitable breeding sites for mosquito development. *An. gambiae* s.l. and *An. funestus* s.l. are the two main malaria vectors found in the village, with *An. funestus* being the most abundant throughout the year exhibiting opportunistic feeding and resting behaviour coupled with a very high malaria transmission intensity (792 infective bites/person per year (ib/p/y)) [7]. *An. funestus* populations from Mibellon displayed a high resistance intensity to all the pyrethroids which has led to a complete loss of pyrethroid-based LLINs efficacy [9, 44].

Elende is a village situated in central Cameroon close to Yaoundé and about 2 km from the Yaoundé-Nsimalen International Airport. This village is characterized by a classical Guinean equatorial climate with four distinct seasons: a short rainy season from mid-March to the end of June; a short dry season from late June to mid-August; a long rainy season that runs from mid-August to mid-November and a long dry season which runs from mid-November to mid-March [45]. Some parameters, such as the strong human influence on the vegetation and the closeness of the village to marshy lands and streams joining major rivers, create temporary and permanent breeding sites for malaria vectors such as *Anopheles gambiae* s.l and *An. funestus* s.l. This latter representing the predominant malaria vectors in this site, supporting high yearly transmission intensities both indoor (1116 ib/p/y) and outdoor (468 ib/p/y) [7]. Pyrethroid-based LLINs are the main prevention method with a coverage of around 60% [45].

### Mosquito sampling

From February to September 2021, wild resting female *Anopheles* mosquitoes (F0) were collected in Mibellon and Elende after obtaining verbal consent from household owners. Collections were conducted between 6:00 and 11:00 a.m. by aspirating mosquitoes from indoor walls using a Prokopack electric aspirator (John W. Hook, Gainesville, FL, USA). Following collection, mosquitoes were placed in paper cups covered with untreated mosquito netting and transported to the insectary at the Centre for Research in Infectious Diseases (CRID) in Yaoundé. During transport, cotton wool lightly soaked in sugar solution was provided on top of the cups to serve as a sugar source for the adult mosquitoes.

### Insecticide susceptibility assays

Susceptibility testing using the WHO tube bioassay [8] was carried-out on the indoor resting wild-caught (F_0_) female *An. funestus* mosquitoes (fed and unfed) from both sites after morphological identification using the keys of Gillies & Coetzee, 1987 [46]. Captured females, mainly blood fed were allowed to digest their blood meal for 2 to 3 days before all collected mosquitoes be exposed to diagnostic dose (DD) of type I [permethrin (0.75%)] and type II [deltamethrin (0.05%) and alpha-cypermethrin (0.05%)] pyrethroid insecticides. Bioassays were conducted under standard insectary conditions of 25 °C (± 2 °C) temperature and 80% (± 10%) relative humidity. For each test, four replicates of 20–25 female F_0_ mosquitoes were exposed to insecticide-impregnated papers for 1 h. After exposure, the mosquitoes were transferred to a holding tube containing cotton soaked in a 10% sugar solution. Mortality was assessed 24 h later. For each bioassay, control tubes containing non-impregnated papers were used.

Based on the results of resistance status with 1 × (DD) of pyrethroids, intensity bioassays were carried out with 5 × and 10 × DD of permethrin (3.75% and 7.5%) deltamethrin (0.25% and 0.5%) and alpha-cypermethrin (0.25% and 0.5%). The intensity bioassays with 5 × and 10 × DD were performed following the WHO 2016 test procedure [8]. Synergist tests with piperonyl butoxide (PBO 4%, a cytochrome P450s inhibitor) and diethyl maleate (DEM 8%, a glutathione-S-transferases (GSTs) inhibitor) were performed to determine the possible involvement of cytochrome P450s and GSTs in the observed resistance.

### DNA extraction and molecular identification of anopheline species

Genomic DNA was extracted from the head–thorax and abdomen of both resistant and susceptible F_0_ mosquitoes using the Livak protocol [47]. Individual mosquitoes were homogenized in pre-heated Livak buffer, followed by cell lysis and incubation at 65 °C. Proteins and cellular debris were removed by potassium acetate precipitation and centrifugation. DNA was precipitated with absolute ethanol, washed with 70% ethanol, air-dried, and resuspended in sterile water. DNA concentration and purity were assessed using a NanoDrop spectrophotometer, and samples were stored at − 20 °C until further molecular analyses.

Molecular identification of species belonging to the *Anopheles funestus* group was performed using genomic DNA previously extracted. Species identification was carried out using the multiplex “Cocktail funestus” PCR assay targeting the ITS2 region of ribosomal DNA, as originally described by Koekemoer et al*.* [48]. This assay combines universal ITS2 primers with species-specific primers designed to discriminate members of the *An. funestus* complex (*An. funestus s.s., An. vaneedeni, An. rivulorum, An. leesoni, An. parensis* and *An. rivulorum-like*). PCR products were separated by electrophoresis on a 1.5% agarose gel, and species were identified based on diagnostic fragment sizes.

### Estimation of plasmodium infection rates

Estimation of *Plasmodium* infection rates was performed using genomic DNA extracted from the head–thorax of indoor-resting *Anopheles* mosquitoes. Initial detection of *Plasmodium* infections was conducted using a real-time TaqMan PCR assay following the protocol of Bass et al*.* [49], targeting the small subunit ribosomal RNA (ssrRNA) gene. This assay allows simultaneous detection of *Plasmodium falciparum* and the *P. ovale / P. vivax/P. malariae* group using species-specific primers and fluorescent probes. Reactions were run on an MX3005P real-time PCR platform, and fluorescence signals were used to identify single or mixed infections.

All TaqMan-positive samples were subsequently subjected to nested PCR following the protocol of Snounou et al*.* [50] to confirm infection and discriminate *Plasmodium* species. This method involved two successive amplification rounds of the ssrRNA gene, using genus-specific primers in the first round and species-specific primers in the second round. Minor modifications were introduced, including the replacement of AmpliTaq polymerase with KapaTaq and the implementation of a multiplex species-detection strategy during the second amplification [7, 51]. PCR products were visualized on 1.5% agarose gels, and species identification was based on diagnostic fragment sizes.

### Genotyping of metabolic resistance markers in *An. funestus* s.s.

Genotyping of metabolic resistance markers in *Anopheles funestus* s.s*.* was performed using allele-specific PCR (AS-PCR) to detect the L119F mutation in the *GSTe2* gene and the G454A mutation in the *CYP9K1* gene as described by Tchouakui et al*.* [35] and Djoko et al*.* [21] respectively. For each locus, AS-PCR relied on the combined use of external primers amplifying the target region and internal primers specific to the susceptible and resistant alleles. PCR reactions were performed in a final volume of 15 µl containing genomic DNA, locus-specific primers, dNTPs, MgCl₂, reaction buffer, and Taq polymerase. Amplifications included an initial denaturation step followed by repeated cycles of denaturation, annealing, and extension, and a final extension step. PCR products were separated by electrophoresis on 1.5% agarose gels and visualized using Midori Green staining. Genotypes were assigned based on the presence or absence of allele-specific amplification products, allowing classification of specimens as homozygous susceptible, heterozygous, or homozygous resistant.

### Statistical analysis

Pearson’s Chi-squared test and odds ratio analyses were used to assess associations between phenotypic resistance, genotypic resistance, and mosquito infection status, using GraphPad Prism (version 8.0.2) and OriginPro 2021 (version 9.8.0.200). When expected cell counts met standard assumptions, Chi-squared tests were applied as the primary analytical approach. For comparisons involving small expected counts (< 5), results were verified using Fisher’s exact test. Statistical significance was set at *p* < 0.05.

## Results

### Species composition of field collected mosquitoes

**Fig. 2:**
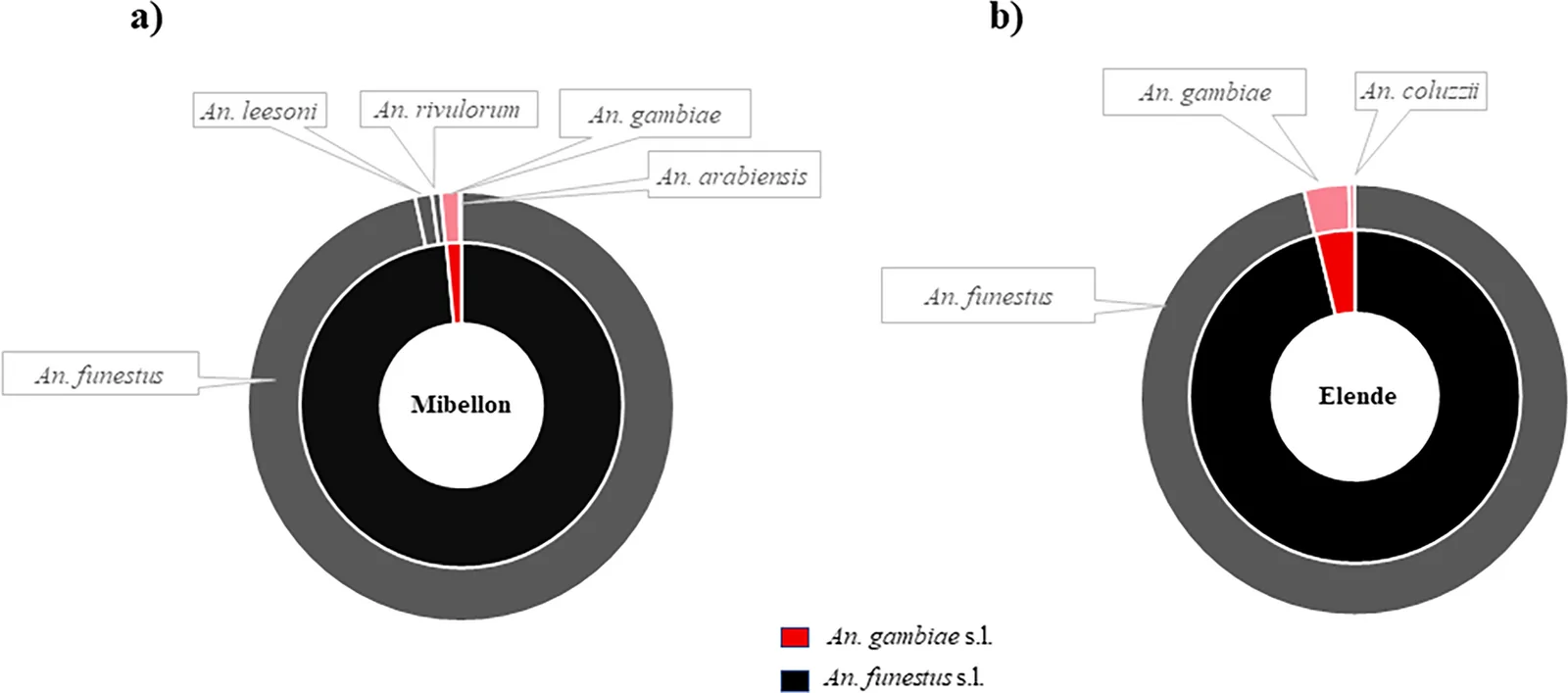
Vector species composition and molecular identification of anopheline species after intra- domiciliary collections. **a** Mibellon; **b** Elende

The indoor collection in Mibellon resulted in about 4250 mosquitoes of *Anopheles* species, with the *An. funestus* group accounting for 98.5% [4186/4250], followed by the *An. gambiae* complex (1.5% [64/4250]) (Fig. 2a). A similar trend was observed in Elende (*An. funestus* s.l.: 96.2% [1270/1320]; *An. gambiae* s.l.: 3.8% [50/1320]) (Fig. 2b). In Mibellon, molecular identification indicated that *An. funestus* s.s. (147/150 [98%]), *An. leesoni* (2/150 [1.3%]) and *An. rivulorum* (1/150 [0.7%]) were members of the *An. funestus* group, whereas *An. gambiae* s.s. (47/50 [94%]) and *An. arabiensis* (3/50 [6%]) were identified as members of the *An. gambiae* complex (Fig. 2a). *An. funestus* s.s (100/100 [100%]) was the only species of the *An. funestus* group in Elende, whilst the *An. gambiae* complex was made up of *An. gambiae* s.s. (40/45 [89%]) and *An. coluzzii* (5/45 [11%]) (Fig. 2b).

### Insecticide susceptibility assays

#### Bioassays with the discriminating dose (DD, 1x)

**Fig. 3:**
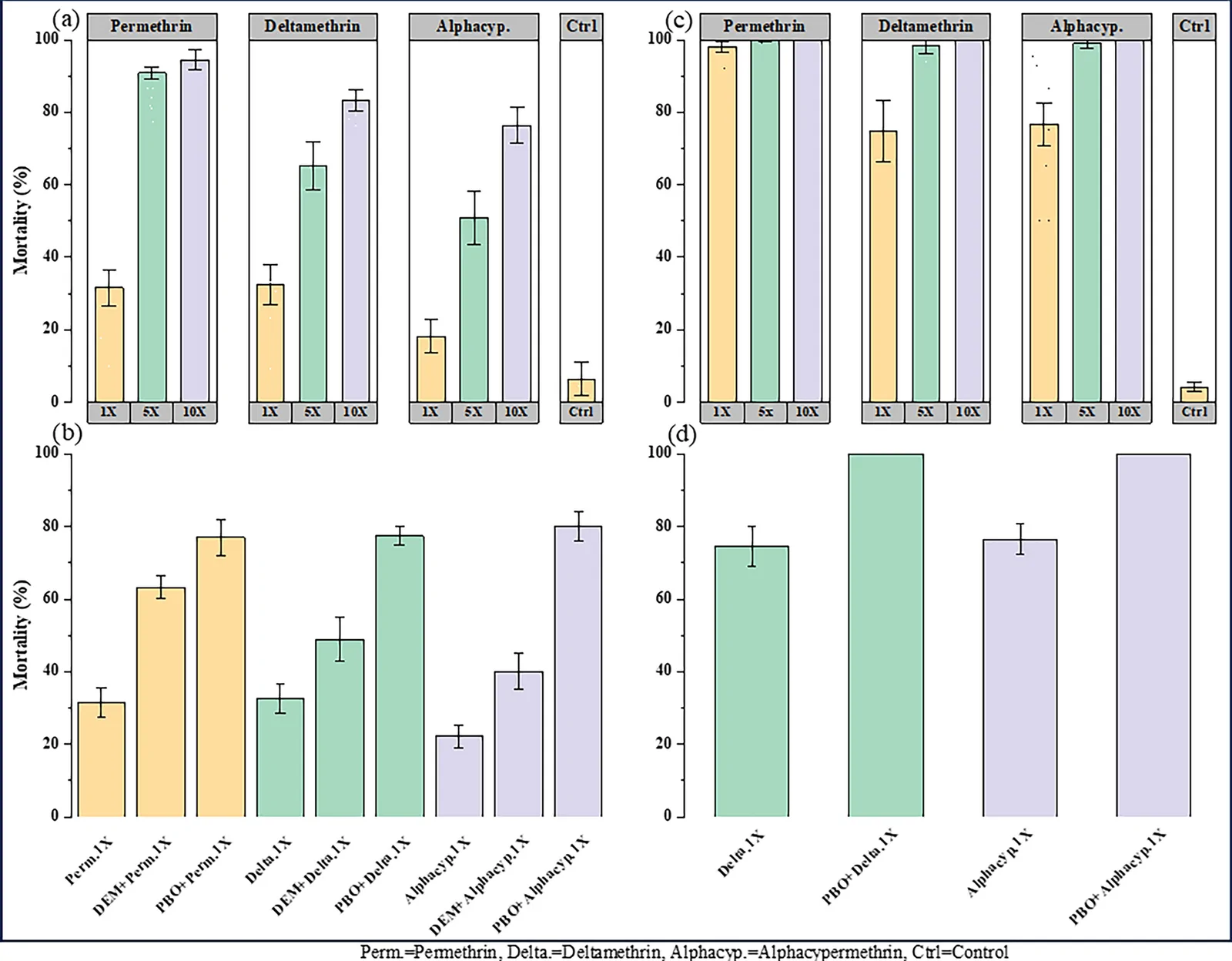
Susceptibility profile of wild *Anopheles funestus* s.l. (F_0_) to pyrethroids insecticides in Mibellon and Elende. **a** Resistance intensity tests in Mibellon; **b** synergist tests with PBO and DEM in Mibellon; **c** resistance intensity tests in Elende; **d** synergists tests with PBO in Elende

In Mibellon, low mortality rate (MR) was observed after exposure of wild-caught (F_0_) *An. funestus* s.l. mosquitoes to the DD of the three pyrethroid insecticides used, permethrin (1x, n = 200: MR = 31.6% ± 3.4%), deltamethrin (1x, n = 185: MR = 32.5% ± 4%) and alpha-cypermethrin (1x, n = 168: MR = 18.24% ± 3.1%) (Fig. 3a). Conversely, in Elende resistance was only observed to the DD of the type II pyrethroids, deltamethrin (1x, n = 200: MR = 74.7% ± 5.6%) and alpha-cypermethrin (1x, n = 183: MR = 76.6% ± 3.1%), while F_0_ *An. funestus* s.l. mosquitoes were susceptible to the type I permethrin (1x, n = 200: MR = 98.8%) (Fig. 3c). This shows that the level of pyrethroids resistance is higher in *An. funestus* from Mibellon than in Elende.

#### Bioassays with pyrethroid 5 × and 10 × DD

Due to the low mortality observed at the discriminating dose (DD) of pyrethroids in the F_0_ anopheline population from Mibellon, higher concentrations of 5 × and 10 × DD of these insecticides were tested to determine the pyrethroid resistance intensity. These tests induced significantly higher mortality rates (MR) compared to the 1 × dose of the three pyrethroids tested [permethrin (5x, n = 260: MR = 90.8% ± 1.1%, *p* < 0.0001; 10x, n = 350: MR = 94.4% ± 2%, *p* < 0.0001); deltamethrin (5x, n = 233: MR = 65.1% ± 4.5%, *p* < 0.001; 10x, n = 322: MR = 83.4% ± 2%, *p* < 0.0001); alpha-cypermethrin (5x, n = 212: MR = 50.8% ± 5%, *p* < 0.001; 10x, n = 295: MR = 76.3% ± 3.3%, *p* < 0.0001)]. However, despite this significant increase in the observed mortality rates, the threshold of 98% recommended by the WHO was not reached for the 10 × dose (Fig. 3 a), indicating a high intensity of pyrethroids resistance or resistance escalation or super-resistance in populations of *An. funestus s.l.* (F_0_) from Mibellon. In Elende, on the other hand, wild mosquitoes of the same species were completely susceptible to the high doses of the three insecticides used (MR > 98%) (Fig. 3c), confirming a low level of pyrethroid resistance in this locality.

#### Bioassays with the synergist PBO and DEM

PBO pre-exposure of F_0_ *An. funestus* s.l. from Mibellon prior to exposure to the DD of pyrethroid insecticides significantly increased mosquito mortality compared to simple exposure to the DD of these pyrethroids: permethrin (1x, n = 200: MR = 31. 6% ± 3.4% vs. PBO + 1x, n = 187: MR = 77% ± 3%; *p* < 0.05), deltamethrin (1x, n = 185: MR = 32.5% ± 4% vs. PBO + 1x, n = 190: 77.4% ± 3%; *p* < 0.005) and alpha-cypermethrin (1x, n = 168: MR = 18.24% ± 3.1% vs. PBO + 1x, n = 182: MR = 80% ± 4%; *p* < 0.0005) (Fig. 3b). This result suggests a partial involvement of P450 enzymes in the resistance observed in this site. Due to partial recovery of susceptibility, another test was carried out using a different synergist (DEM). Pre-exposure of mosquitoes to the diethyl maleate (DEM), a glutathione S-transferase inhibitor also partially restored their susceptibility to pyrethroids: permethrin (1x, n = 200: MR = 31.6% ± 3. 4% vs DEM + 1x, n = 193: MR = 63.3% ± 3%; *p* < 0.05), deltamethrin (1x, n = 185: MR = 32.5% ± 4% vs DEM + 1x, n = 171: MR = 49% ± 3%;* p* > 0.05) and alpha-cypermethrin (1x, n = 168: MR = 22.2% ± 3.1% vs DEM + 1x, n = 157: MR = 40% ± 5%; *p* = 0.04) (Fig. 3b). This result highlights a partial involvement of glutathione S-transferases alongside cytochromes P450 in the observed resistance, which is more pronounced for permethrin than for the other pyrethroids. In Elende, synergist tests were only evaluated with PBO and type II pyrethroids due to the high susceptibility of F_0_ *An. funestus* s.l. to type I insecticide. In contrast to Mibellon, pre-exposure of the mosquitoes to the PBO synergist completely restored their susceptibility to the insecticides used: deltamethrin (1x, n = 200: MR = 74.7% ± 5.6% vs. PBO + 1x, n = 147: MR = 100%; *p* < 0.05), alpha-cypermethrin (1x, n = 183: MR = 76.6% ± 4.1% vs. PBO + 1x, n = 190: MR = 100%; *p* < 0.05) (Fig. 3d). This result confirms a predominant role of P450 enzymes in the resistance observed in this locality.

### Impact of L119F-*GSTe2* and G454A-*CYP9K1* mutations on the escalation of pyrethroid resistance in field *Anopheles funestus* s.s. (F_0_)

#### Association between the L119F-*GSTe2* mutation and pyrethroid resistance escalation in *Anopheles funestus* s.s. (F_0_) from Mibellon

Regarding the high pyrethroid resistance intensity observed in Mibellon, a total of 1366 mosquitoes (680 alive and 686 dead) were genotyped for the L119F-*GSTe2* mutation to determine its role in the resistance escalation in this locality. Of these, 162 mosquitoes (11.9%) were homozygous resistant 119F/F (RR), 739 (54.1%) were heterozygous L119F (RS), and 465 (34.0%) were homozygous susceptible L/L119 (SS). Overall, the frequency of the resistant 119F allele was 39%.

- Impact on permethrin resistance

**Fig. 4:**
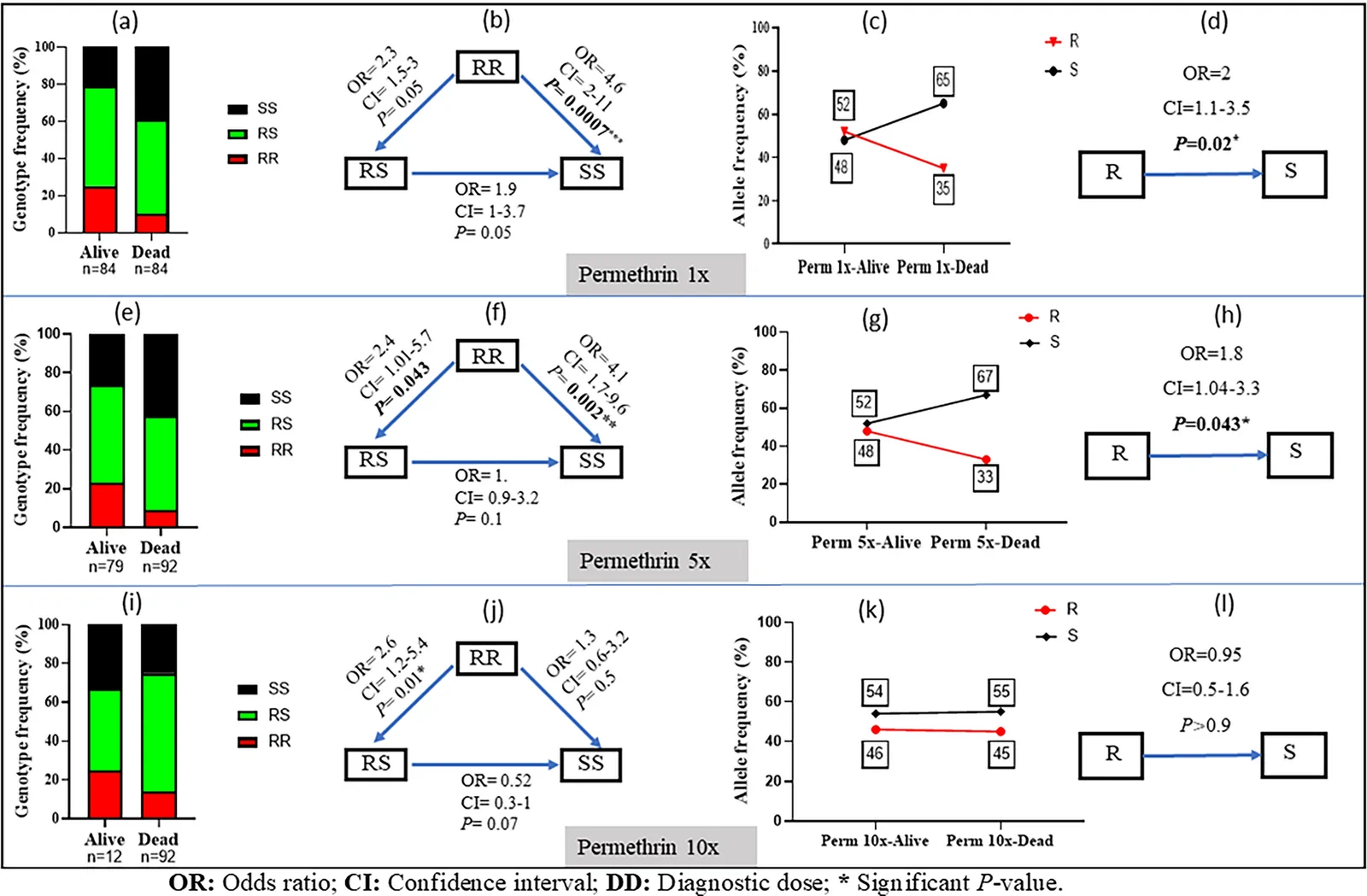
Impact of the L119F-*GSTe2* mutation on permethrin (type I) resistance in *Anopheles funestus* s.s. from Mibellon. Panels **a**, **b** show associations between genotype and survival following exposure to 1 × diagnostic dose (DD) of permethrin at the genotypic level (L/L119-SS, L119F-RS, 119F/F-RR). Panels **c**, **d** present the same analysis at the allelic level (L119 vs 119F). Panels **e**, **f** and **g**, **h** show genotypic and allelic associations following exposure to 5 × DD, respectively, while panels **i**, **j** and **k**, **l** present genotypic and allelic associations following exposure to 10 × DD. Associations between genotype/allele and survival were assessed using Pearson’s Chi-squared and odds ratios (ORs) with 95% confidence intervals (CI). Statistical significance was set at *P* < 0.05

A significant difference in the genotype distribution of this marker was observed between alive and dead mosquitoes after exposure to increasing concentration of permethrin (1 × or DD: χ^2^ = 11.91, *p* = 0.003; 5 × DD: χ^2^ = 9.9, *p* = 0.007 and 10 × DD: χ^2^ = 7.7, *p* = 0.02). Estimation of the odds ratio revealed that mutant mosquitoes (119F/F-RR resistant homozygotes) were significantly more able to survive exposure to permethrin at doses of 1x (OR = 4.6, CI [2.1–11], *p* = 0.0007) and 5 × DD (OR = 4.1, CI [1.7–9.6], *p* = 0.002) compared to the L/L119-SS susceptible homozygous wild-type mosquitoes (Fig. 4 a, b, e, f). Consequently, at the allelic level, mosquitoes carrying the resistant allele 119F had significantly higher ability to survive exposure to permethrin 1x (OR = 2.01, CI [1.1–3.5], *p* = 0.02) and 5x (OR = 1.8, CI [1.04–3.3], *p* = 0.043) than mosquitoes carrying the susceptible allele L119 (Fig. 4 c, d, g, h). This result confirms that the L119F-*GSTe2* mutation is associated with permethrin resistance (1x) and its escalation (5x) in wild population of *An. funestus* s.s. At 10 × DD, no difference was seeing between dead and alive [OR = 1.04, CI (0.6–1.8), *p* > 0.9)] (Fig. 4 k, l) indicating that at a very high dose of insecticide, this resistance marker is not the primary mechanisms allowing mosquitoes survivorship.

b. Impact on deltamethrin resistance

**Fig. 5:**
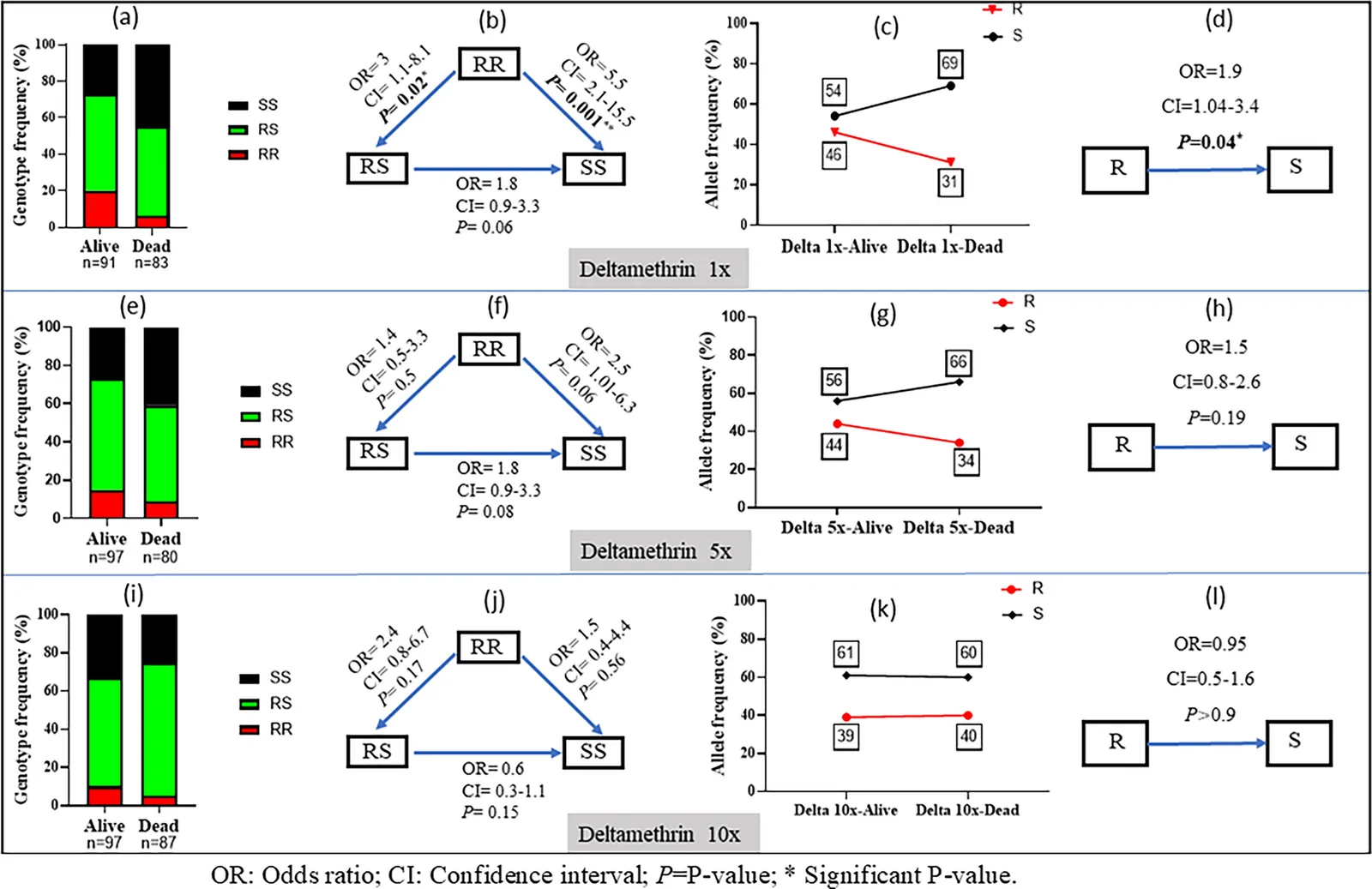
Impact of the L119F-*GSTe2* mutation on deltamethrin (type II pyrethroid) resistance in *Anopheles funestus* s.s. from Mibellon. Panels **a**, **b** show associations between L119F-*GSTe2* genotypes (L/L119-SS, L119F-RS, 119F/F-RR) and survival following exposure to the 1 × diagnostic dose (DD) of deltamethrin at the genotypic level, while panels **c**, **d** present the corresponding allelic-level analysis (L119 vs 119F). Panels **e**, **f** and **g**, **h** show genotypic and allelic associations following exposure to 5 × DD, respectively, and panels **i**, **j** and **k**, **l** show genotypic and allelic associations following exposure to 10 × DD. Associations between genotype/allele and survival were assessed using Pearson’s Chi-squared and odds ratios (ORs) with 95% confidence intervals (CI). Statistical significance was set at *P* < 0.05

After deltamethrin exposure, a significant difference was observed only for the DD (1x: χ^2^ = 11.2, *p* = 0.002) where homozygous resistant 119F/F-RR mosquitoes were significantly more able to survive exposure than homozygous susceptible L/L119-SS [1x: OR = 5.5, CI (2.1–15.5), *p* = 0.001] and heterozygous L119F-RS [1x: OR = 3, CI (1.1–8.1), *p* = 0.02] mosquitoes (Fig. 5 a, b). Consequently, mosquitoes carrying the resistant allele 119F were significantly more likely to survive exposure to this dose than their counterparts carrying the susceptible allele L119 [1x: OR = 1.9, CI (1.04–3.4), *p* = 0.043] (Fig. 5 c, d), confirming the involvement of this marker in the observed resistance. However, the fact that there was no significant difference in the allele distribution of this marker between alive and dead phenotypes after exposure to 5 × and 10 × DD of deltamethrin confirmed that this mutation was not responsible for the deltamethrin resistance escalation observed in Mibellon [5x: OR = 1.5, CI (0.8–2.6), *p* = 0.2; 10x: OR = 0.95, CI (0.5–1.6), *p* > 0.9] (Fig. 5h, l).

c. Impact on alpha-cypermethrin resistance

Concerning the alpha-cypermethrin exposure, a significant difference was only observed for the DD (1x: χ^2^ = 11.2, *p* = 0.002), as noted for deltamethrin although no significant difference was observed at the allelic level [1x: OR = 0.9, CI (0.5–1.7), *p* > 0.9; 5x: OR = 0.8, CI (0.4–1.5), *p* > 0.6; 10x: OR = 1.04, CI (0.6–1.8), *p* > 0.9] (Figure S1). This result shows that this resistance marker is less associated with resistance or resistance escalation to alpha-cypermethrin in Mibellon.

The high frequencies (> 95%) of resistance markers like G454A-*CYP9K1* and the 4.3 kb insertion, combined with the absence of markers such as *CYP6P9a/b* and the *6.5 kb* insertion, hindered the evaluation of their impact on the observed resistance.

####  Association between L119F-*GSTe2*/G454A-*CYP9K1* mutations and pyrethroid resistance in *Anopheles funestus* s.s. (F_0_) from Elende

In Elende, the frequency of the L119F-*GSTe2* mutation was 56% (181 RR, 270 RS, 115 SS), while that of the G454A-*CYP9K1* mutation was 28% (47 454A/A-RR, 229 G454A-RS, 300 G/G454-SS). Fisher's exact test and odds ratio showed no significant difference in the genotypic (χ^2^ = 0.9; *p* = 0.7) and allelic (OR = 0.8; CI [0.5–1.5]; *p* = 0.6) frequency distribution of L119F-*GSTe2* between alive and dead mosquitoes after exposure to the DD of deltamethrin, indicating that this marker is not associated with the observed resistance (Figure S2 a-d). As observed with deltamethrin, this mutation is not associated with the observed resistance to alpha-cypermethrin.

**Fig. 6:**
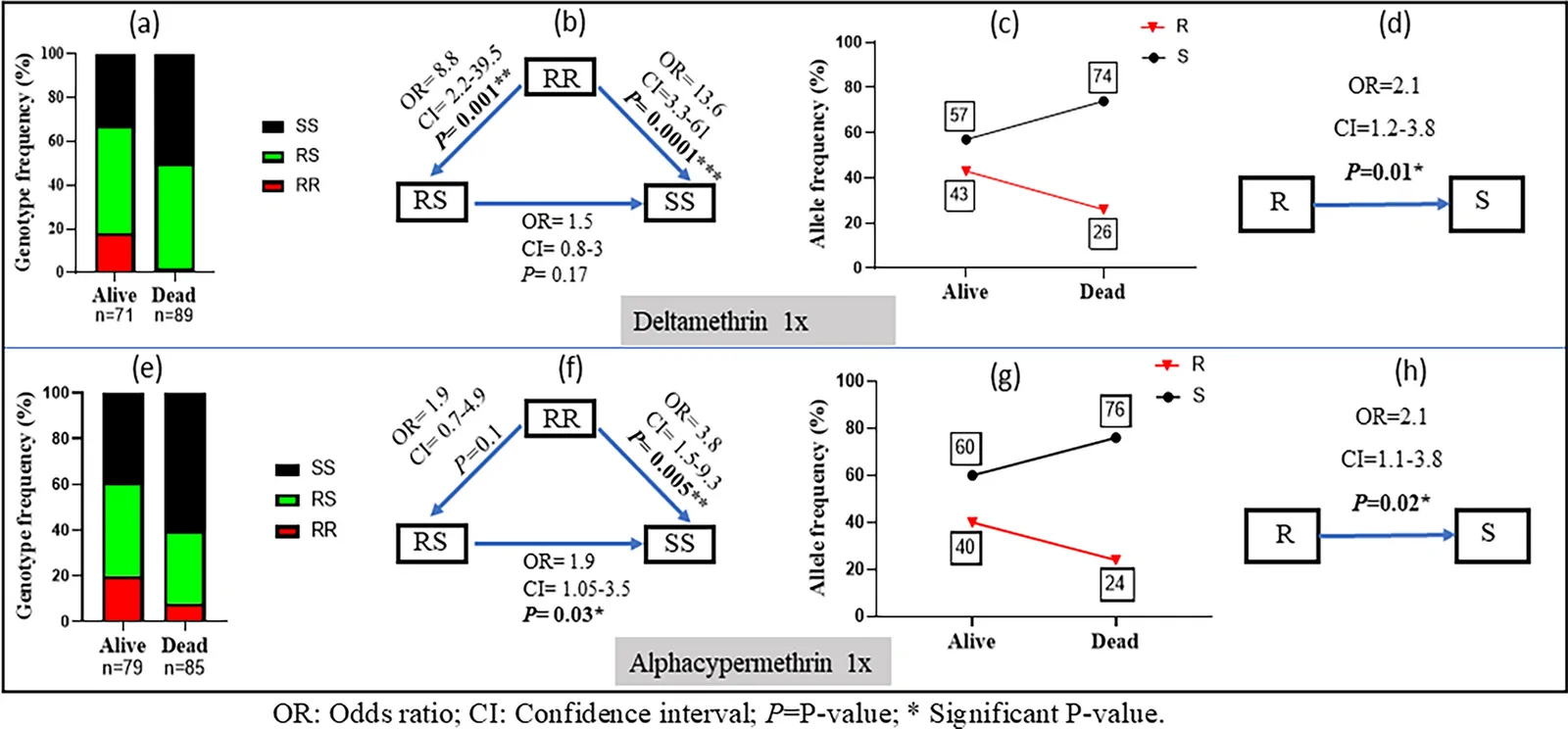
Impact of the G454A-*CYP9K1* mutation on type II pyrethroid (deltamethrin and alphacypermethrin) resistance in *Anopheles funestus* s.s. from Elende. **a**, **b** Deltamethrin 1 × at the genotypic level; **c**, **d** Deltamethrin 1 × at the allelic level; **e**, **f** Alphacypermethrin 1 × at the genotypic level; **g**, **h** Alphacypermethrin 1 × at the allelic level. Panels **a**, **b** show associations between G454A-*CYP9K1* genotypes (G/G454-SS, G454A-RS, A/A454-RR) and survival following exposure to the 1 × diagnostic dose (DD) of deltamethrin at the genotypic level, while panels (**c, d**) present the corresponding allelic-level analysis (G454 vs 454A). Panels **e**, **f** and **g**, **h** show genotypic and allelic associations following exposure to 1 × DD of alphacypermethrin. Associations between genotype/allele and survival were assessed using Pearson’s Chi-squared and odds ratios (ORs) with 95% confidence intervals (CI). Statistical significance was set at *P* < 0.05

In contrast to the results observed with the previous mutation, resistance of field mosquitoes to the DD of deltamethrin and alpha-cypermethrin was strongly associated with the G454A-*CYP9K1* resistance marker. Mosquitoes carrying the resistant 454A-*CYP9K1* allele were significantly more likely to survive exposure to deltamethrin (OR = 2.1; CI [1.2–3.8]; *p* = 0.01) (Fig. 6c, d) and alpha-cypermethrin (OR = 2.1; 95% CI [1.1–3.8]; *p* = 0.02) compared to mosquitoes carrying the susceptible G454-*CYP9K1* allele (Fig. 6g, h).

After characterizing their resistance profiles, different phenotypes of *Anopheles funestus* s.l. (survivors and fatalities from varying insecticide doses) were selected, and their *Plasmodium* spp. infection status was determined to evaluate the effects of pyrethroid resistance escalation on vectorial capacity.

### Relationship between pyrethroid resistance escalation and vectorial capacity of *Anopheles funestus* s.l. (F_0_)

#### Sporozoite infection rate

**Fig. 7:**
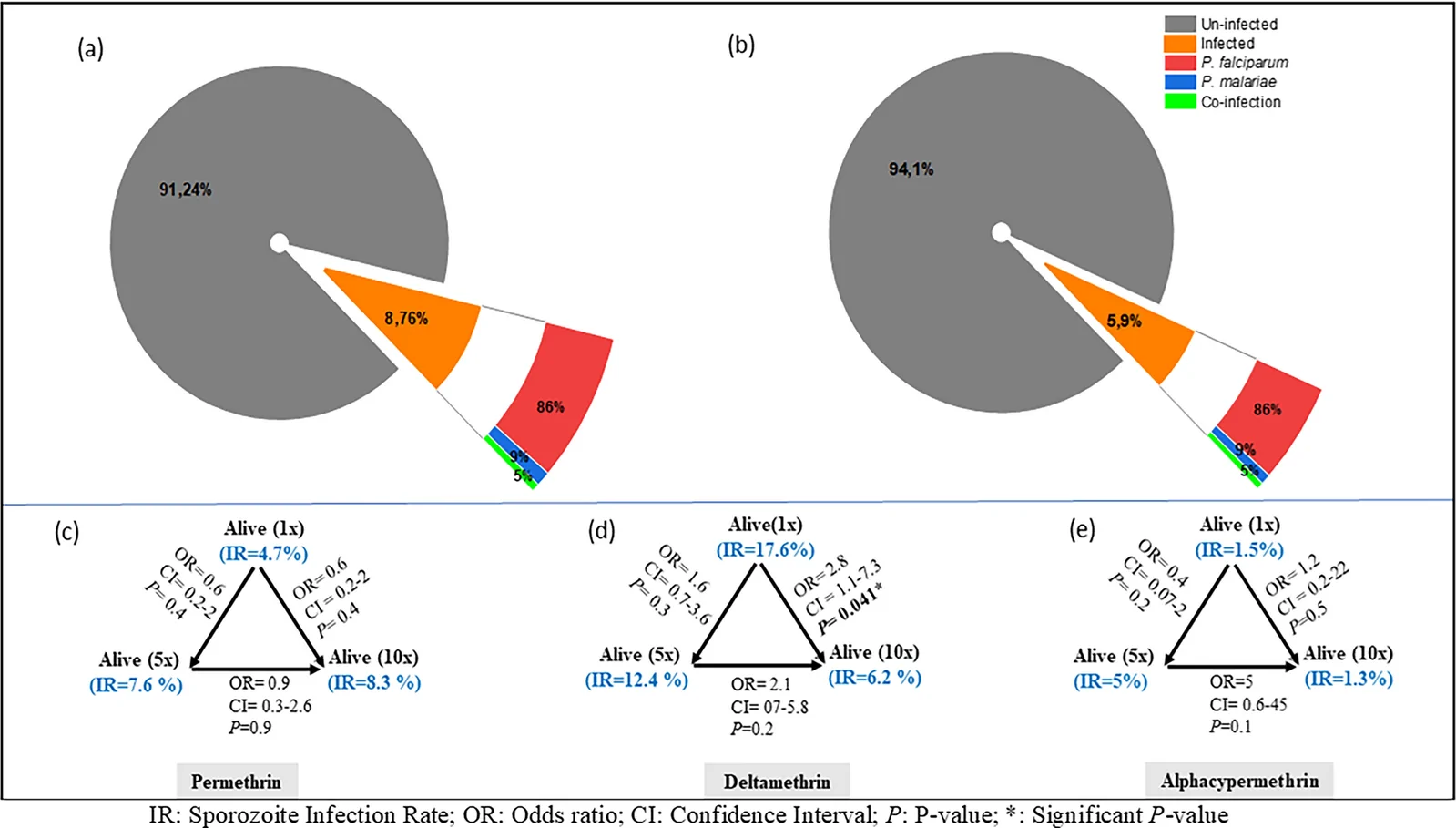
Phenotypic impact of pyrethroids resistance escalation on the vectorial capacity of *Anopheles funestus* s.l. **a** Sporozoite infection rate in Mibellon; **b** Sporozoite infection rate in Elende; **c–e** Contrasting effects of type I (permethrin) and type II (deltamethrin) pyrethroids on the vectorial capacity of wild-caught *Anopheles funestus* s.l.

In the highly resistant *An. funestus* population from Mibellon, a total of 1449 samples (head-thorax) from the different phenotypes (1x: dead = 219, alive = 253; 5x: dead = 268, alive = 271; 10x: dead = 240, alive = 198) were used to determine the infection rate by *Plasmodium* sporozoites. Of these samples, 127 were infected, giving an overall sporozoite infection rate (SIR) of 8.76%. Of the *Plasmodium* species identified, *P. falciparum* was the predominant species, accounting for 86% (n = 110) of the total SIR observed, followed by *P. malariae* (9%, n = 11) and *P. falciparum-P. malariae* co-infection (5%, n = 6) (Fig. 7a). In lowly resistant population from Elende, of the 576 samples tested (dead = 300, alive = 276), 34 mosquitoes were infected, giving an overall SIR of 6%. As previously observed, *P. falciparum* was the predominant species accounting for 85% (n = 29) of the total SIR, followed by *P. malariae* (9%, n = 3) and *P. falciparum-P. malariae co-infection* (6%, n = 2) (Fig. 7b).

#### Phenotypic association: link between phenotypic resistance escalation and vectorial capacity of *Anopheles funestus* s.l. (F_0_)

In Mibellon, no significant difference was observed between the SIRs of alive and dead mosquitoes regardless of the permethrin insecticide concentration used (*p* > 0.05, Table 1). Conversely, at higher doses (10 × DD) of type II pyrethroids (deltamethrin and alphacypermethrin), dead mosquitoes were significantly more infected than alive mosquitoes (*p* < 0.05, Table 1), showing a high efficacy of type II pyrethroids against *Plasmodium-*infected mosquitoes. Regarding field mosquitoes surviving exposure to different doses of permethrin, although no significant difference was observed (χ^2^ = 0.6; *p* = 0.7), SIRs were higher in mosquitoes surviving exposure to the higher doses (5 × and 10x) of insecticide (1 × or DD: 4/84 [4. 7%], 5 × DD: 6/79 [7.6%] and 10 × DD: 1/12 [8.3%]) (Fig. 7c), indicating that the vectorial capacity of F_0_ mosquitoes still increased with the level of permethrin (type I pyrethroid) resistance. Similarly, no significant difference (χ^2^ = 4.6; *p* = 0.09) was observed between the SIRs of mosquitoes surviving exposure to different doses of deltamethrin (1x: 16/91 [17.6%]), 5 × DD (12/97 [12.4%]) and 10 × DD (6/97 [6.2%]). However, odds ratio (OR) analysis showed that there was a significant reduction in the vectorial capacity of mosquitoes surviving exposure to the higher doses of deltamethrin (5 × and 10 × DD) compared to those surviving DD (1x) (OR = 2.8; 95% confidence interval (CI) = [1.1–7.3]; *p* = 0.041) (Fig. 7d). Together with the previous observation that dead mosquitoes exposed to a higher dose of type II pyrethroid were significantly more infected than surviving mosquitoes, this underscores the fact that type II pyrethroids may be best suited to control wild infected resistant mosquitoes (F0).

**Table 1:** Sporozoite infection rate between alive and dead mosquitoes after exposure to different doses of pyrethroids insecticides in Mibellon and Elende

Insecticides	Insecticide concentrations	Phenotypes	Sporozoite infection			Chi-square test	
			Tested	Positive	% Infection	*x*^2^	*P*-value
			Mibellon				
Permethrin	Pem.1x	Alive	84	4	4.7	2.7	0.2
		Dead	82	9	11		
		Total	166	13	7.8		
	Perm.5x	Alive	79	6	7.6	2.7	
		Dead	89	2	2.2		0.1
		Total	168	8	4.8		
	Perm.10x	Alive	12	1	8.3	0.5	0.5
		Dead	77	3	3.8		
		Total	89	4	4.5		
Deltamethrin	Delta.1X	Alive	91	16	17.5	0.3	0.6
		Dead	83	12	14.5		
		Total	174	28	16.1		
	Delta.5X	Alive	97	12	12.4	1.86	
		Dead	80	5	6.3		0.2
		Total	177	17	9.6		
	Delta.10X	Alive	97	6	6.2	0.8	0.03*
		Dead	87	13	15		
		Total	184	19	10.3		
Alphacypermethrin	Alpha.1X	Alive	64	1	1.5	2.3	0.1
		Dead	40	3	7.5		
		Total	104	4	3.8		
	Alpha.5X	Alive	81	4	5	0.3	0.6
		Dead	85	6	7		
		Total	166	10	6		
	Alpha.10X	Alive	75	1	1.3	5.1	0.02^*^
		Dead	60	6	10		
		Total	135	7	5.2		
			**Elende**				
Deltamethrin	Delta. 1x	Alive	71	1	1.4	0.1	0.6
		Dead	89	2	2.2		
		Total	160	3	1.8		
Alphacypermethrin	Alpha. 1x	Alive	56	3	5.3	0.1	0.8
		Dead	89	4	4.5		
		Total	145	7	4.8		

Due to the low level of resistance observed in Elende (no survivors at 5 × and 10 × DD of pyrethroids), comparisons were only made between surviving and dead mosquitoes after exposure to the DD of deltamethrin and alpha-cypermethrin. No significant difference was observed between the SIRs of these mosquitoes after exposure to these insecticides (χ^2^ = 0.1; *p* = 0.6; χ^2^ = 0.05; *p* = 0.8) (Table 1).

#### Genotypic association: link between L119F-*GSTe2*/G454A-*CYP9K1* mutations and vectorial capacity of *Anopheles funestus* s.s. (F_0_)

- Effect of the L119F-*GSTe2* mutation

A significant difference was observed in the distribution of the L119F-*GSTe2* genotypes between infected and uninfected wild mosquitoes in Mibellon (χ^2^ = 8.2; *p* = 0.01) (Table 2). Indeed, homozygous resistant 119F/F-RR (OR = 2.1; 95%IC = [1.1–4]; *p* = 0.03) and heterozygous L119F-RS (OR = 1.9; 95%IC = [1.2–3]; *p* = 0.006) mosquitoes were significantly more infected with *Plasmodium* spp. sporozoites than their homozygous susceptible L/L119-SS counterparts (Table 3). This result indicates that the L119F-*GSTe2* mutation, while associated with permethrin resistance and its exacerbation, also significantly enhances the vectorial capacity of *An. funestus* s.s. in Mibellon (Adamaoua region). The amplifying effect of this mutation on the infectivity of *An. funestus* was confirmed in Elende (Centre region), where homozygous resistant mosquitoes were approximately three times more likely to be infected than their homozygous susceptible counterparts (OR = 3.3; 95% CI [1.05–11]; *p* = 0.048) (Table 3). This underscores the strong impact of this resistance marker on the ability of *An. funestus* s.s. to host *Plasmodium* spp. and progress to the infective stage under field conditions.

**Table 2:** Impact of L119F-*GSTe2* and G454A-*CYP9K1* metabolic resistance markers on vectorial capacity of wild-caught *Anopheles funestus* s.s. mosquitoes (Fisher Exact probability test based on the proportions)

Sites	Sporozoite infection	Abundance	L119F-*GSTe2* genotypes			Chi-square test	
			RR (%)	RS (%)	SS (%)	*X*^2^	*P*-value
Mibellon	Infected	114	17(15)	72 (63)	25 (22)	8.2	0.01*
	Non infected	1252	145 (12)	667 (53)	440 (35)		
	Total	1366	162	739	465		
	Infection %	8.3	10.5	9.7	5.4		
Elende	Infected	34	15 (44)	16 (47)	3 (9)	4	0.1
	Non infected	532	166 (31)	254 (48)	112 (20)		
	Total	566	181	270	115		
	Infection %	6	8.2	6	2.6		
			**G454A-*CYP9K1* genotypes**				
Elende	Infected	34	1 (3)	13 (38)	20 (59)	2.7	0.2
	Non infected	542	46 (8)	216 (40)	280 (52)		
	Total	576	47	229	300		
	Infection %	6	2.1	5.6	6.6

**Table 3:** Impact of L119F-*GSTe2* and G454A-*CYP9K1* metabolic resistance markers on vectorial capacity of wild-caught *Anopheles funestus* s.s. mosquitoes (Odds ratio)

Sites	L119F-*Gste2* Genotypes	OR	CI	*P*-value
Mibellon	RR vs. RS	1.08	0.6–1.8	0.7
	RR vs. SS	2.1	1.1–4	0.03*
	RS vs. SS	1.9	1.2–3	0.006**
	R vs S	1.4	0.8–2.5	0.2
Elende	RR vs. RS	1.4	0.7–3	0.8
	RR vs. SS	3.3	1.05–11	0.048*
	RS vs. SS	2.3	0.7–8	0.2
	R vs S	1.7	0.9–3.1	0.1
	**G454A-*CYP9K1* Genotypes**			*P*
Elende	RR vs. RS	0.4	0.2	0.1–1.5
	RR vs. SS	0.3	0.1	0.1–1.2
	RS vs. SS	0.8	0.5	0.5–1.5
	R vs S	0.7	0.4	0.4–1.4

b. Effect of the G454A-*CYP9K1* mutation

The study of the effect of the G454A-*CYP9K1* resistance marker on the vectorial capacity of *An. funestus* s.s. in Elende showed an opposite trend to that observed with L119F-*GSTe2*. Although no significant difference was observed in the distribution of its genotypes between infected and uninfected mosquitoes (χ^2^ = 2.7; *p* = 0.2), G/G454-SS homozygous susceptible and G454A-RS heterozygous mosquitoes were more infected than 454A/A-RR homozygous resistant mosquitoes (Table 2). However, the odds ratio test showed no significant association between this mutation and the mosquito infectious status (Table 3).

## Discussion

Malaria control is under constant threat from the development and escalation of insecticide resistance in major vectors in Africa. Their impact on the efficacy of vector control tools is well documented, but little is known about their effect on the *Plasmodium* spp. infection/transmission. In this study, having established their susceptibility profile to pyrethroid insecticides, we assessed the impact of pyrethroid exposure, resistance escalation and metabolic resistance (L119F-*GSTe2* and G454A-*CYP9K1)* on the survivorship and vectorial capacity of *Plasmodium*-infected wild *An. funestus* (F_0_) mosquitoes. The study highlighted the predominant epidemiological role of wild-caught *An. funestus* s.l.; the high efficacy of type II pyrethroids against *Plasmodium*-infected resistant *Anopheles funestus* s.l.; sign of an association between pyrethroid resistance escalation and an increased likelihood of *Plasmodium* spp. transmission; and a significant role of the L119F-*GSTe2* mutation in pyrethroid resistance escalation and *Plasmodium* transmission risk.

### *Anopheles funestus* s.l., the dominant vector species at both collection sites, plays a significant epidemiological role

The indoor collections at both sites predominantly consisted of *An. funestus* s.l*.*, confirming its status as the dominant vector in some areas, even when coexisting with other important malaria vectors. This finding aligns with previous studies that emphasize the unique ecological and behavioral traits of *An. funestus*, particularly its high levels of endophily (indoor-resting) and anthropophily (human-biting) across various ecoclimatic regions in Cameroon [7, 45, 52] but also in eastern [53, 54] and southern Africa [55, 56]. Their strong endophilic behavior observed in this study, surpassing that of other malaria vectors, greatly enhances the mosquito's likelihood of contact with humans. This is consistent with previous observations at these sites, where *An. funestus* was associated with an exceptionally high human blood index (94%) [7], reinforcing its role as a highly efficient malaria vector.

The sporozoite infection rate of wild *An. funestus* (F_0_) populations at both sites was high and comparable to previously reported rates [7, 35, 45, 57], with *P. falciparum* overwhelmingly predominant, followed by *P. malariae*. These findings reaffirm the significant epidemiological role of *An. funestus* in Cameroon and underscore the urgency of designing species-specific interventions. Beyond its marked anthropophily and endophily, this vector’s epidemiological importance has been attributed to its preference for permanent or semi-permanent aquatic habitats (persisting beyond the rainy season), its adaptive resting and feeding behaviors (including outdoor and occasional daytime activity), and its pyrethroid resistance profile [7, 12, 32, 54, 56, 58]. The stark disparity in *P. falciparum* and *P. malariae* sporozoite rates may reflect differences in parasite biology relative to mosquito lifespan. *Plasmodium malariae*’s extended sporogonic cycle (21 days versus *P. falciparum*’s 14 days) likely reduces its detection in wild mosquitoes, whose average lifespan rarely exceeds three weeks [57]. Nevertheless, the persistent detection of *P. malariae* at both sites and across Cameroon highlights the need for malaria control programs to prioritize this parasite in elimination strategies, alongside *P. falciparum*.

### *Anopheles funestus* (F_0_) from Mibellon exhibits escalated pyrethroid resistance, contrasting with lower resistance intensity in Elende.

Populations of *Anopheles funestus s.l.* from Mibellon exhibited resistance to permethrin, deltamethrin, and alphacypermethrin across all concentrations (1 × , 5 × , and 10 ×). While this study is the first to demonstrate high-intensity pyrethroid resistance or "super-resistance" in wild *An. funestus* s.l. (F_0_) in Cameroon, similar resistance patterns have been reported previously in first-generation (F_1_) mosquitoes of the same species in Uganda [12], Malawi [59], Congo-Kinshasa [11] and most recently in Cameroon [9]. However, the escalation of pyrethroid resistance in wild (F_0_) *An. funestus* has been infrequently documented until the recent study by Kumala et al*.* [56] in Malawi, which aligns with our findings.

Factors contributing to this resistance include the age of mosquitoes [60] and prior exposure to insecticides in field conditions [8], which can lead to either underestimating or overestimating resistance levels. Additionally, blood meals have been shown to enhance female mosquitoes' ability to tolerate higher insecticide doses [61]. This is likely due to the upregulation of detoxification genes, such as cytochrome P450s, in response to blood feeding, as demonstrated in *Culex pipiens* [62] and *Aedes aegypti* [63]. It is therefore suggested that, in *An. funestus*, detoxification processes aimed at neutralizing toxic blood components may inadvertently increase their capacity to withstand insecticide exposure [61]. This hypothesis is supported by a recent study showing that blood-fed *An. funestus* females exhibited increased longevity and higher levels of pyrethroid resistance, even in older age groups [64]. Despite the challenges of using wild mosquitoes for resistance assessment, they provide insights into real-world conditions affecting vector control interventions on the field, enabling a better understanding of any changes in intervention efficacy. Therefore, while standardized protocols using mosquitoes of a specific age and physiological status remain essential for resistance assessment, quantifying resistance in older, wild-caught females is equally valuable.

In contrast, *An. funestus* s.l. (F_0_) populations from Elende were susceptible to the discriminating dose (DD) of permethrin and exhibited low-intensity resistance to deltamethrin and alphacypermethrin. This difference in resistance patterns between the two sites likely reflects varying levels of insecticide selection pressure, influenced by differences in the history of bed net use and agricultural pesticide application. A notable example of this differential selection pressure is the recent detection of the G454A-*CYP9K1* mutation, which is strongly associated with pyrethroid resistance. This mutation is fixed in the Mibellon population (all mosquitoes carry the resistance allele), whereas many mosquitoes in Elende still carry the susceptible allele [21]. These findings underscore the importance of tailoring vector control strategies to local resistance profiles and selection pressures.

### The G454A-*CYP9K1* and L119F-*GSTe2* mutations are associated to resistance and resistance escalation to pyrethroids, respectively

In Mibellon, genotyping of the L119F-*GSTe2* mutation revealed its association with permethrin and deltamethrin resistance, as well as moderate resistance intensity or resistance escalation at 5 × the diagnostic dose (DD) of permethrin. These findings align with prior studies demonstrating that overexpression of the *GSTe2* gene alone confers resistance to DDT and cross-resistance to pyrethroids [17, 65], with the 119F-*GSTe2* resistant allele metabolizing permethrin more efficiently than deltamethrin [17]. A recent study in Mibellon further confirmed the strong association between L119F-*GSTe2* genotypes and permethrin resistance phenotypes in F_1_ *An. funestus* populations exposed to escalating pyrethroid doses [9]. The mechanism by which *GSTe2* confers pyrethroid resistance may involve direct insecticide metabolism by the mutant enzyme [17, 66] or alternative pathways such as sequestration of toxins or protection against pyrethroid-induced oxidative stress [67]. However, the lack of association between L119F-*GSTe2* and resistance escalation at 10 × DD in Mibellon suggests the involvement of additional resistance mechanisms.

A key limitation of this study is the absence of analyses exploring the combined effects of insecticide resistance markers, particularly given the fixation (high prevalence of resistant alleles) or absence of certain markers in the population. For example, the G454A-*CYP9K1* mutation and *4.3 kb* structural variant, both known to compromise pyrethroid-based net efficacy, are fixed in Mibellon. Knowing that super-resistance in mosquitoes is multifactorial, further limitations include the lack of expression profiling of pyrethroid resistance-associated genes in *An. funestus* s.s., as well as analyses of epicuticle thickening and cuticular hydrocarbon (CHC) content. These factors, combined with the fixation of resistance markers, could collectively explain the observed resistance escalation as recently elucidated [68]. Overall, this highlights the need for further comprehensive molecular studies to elucidate the underlying mechanisms of this super-resistance in wild mosquitoes in order to prevent the failure of future pyrethroid-based interventions in Mibellon.

In Elende, the recently identified G454A-*CYP9K1* mutation was associated with resistance to deltamethrin and alpha-cypermethrin. This finding corroborates earlier data from the same site, where this mutation was linked to reduced efficacy of alpha-cypermethrin-treated nets [21], positioning it as a potential threat to next-generation vector control tools.

### Type II pyrethroids insecticides negatively affect *Plasmodium* infection in resistant *Anopheles funestus* contrasting with type I pyrethroid

Despite the intense pyrethroid resistance observed in Mibellon, our findings demonstrate that exposure to type II pyrethroids, particularly deltamethrin and alphacypermethrin, remains effective against wild *Plasmodium*-infected *An. funestus* mosquitoes. This efficacy may stem from the intrinsic mode of action of type II pyrethroids, which feature an α-cyano group in their chemical structure. This group enhances their binding affinity to voltage-gated sodium channels (VGSCs), leading to more severe and prolonged nerve depolarization compared to type I pyrethroids [69]. This heightened neurotoxic effect may also induce elevated levels of oxidative stress, which could synergize with reactive oxygen species (ROS) or oxidative stress generated by heavy *Plasmodium* infection, pathogen replication, and the associated immune response [70]. Such synergy could exacerbate oxidative damage in mosquitoes exposed to type II pyrethroids, ultimately resulting in higher mortality rates among heavily infected individuals.

One limitation of this study is the use of field-collected mosquitoes with varying physiological statuses and ages. For instance, the observation that dead mosquitoes were significantly more infected could be attributed to their age, as older mosquitoes in field conditions are more likely to harbor infections and may exhibit increased susceptibility to insecticides. To address this limitation, future studies will involve rearing field-collected mosquitoes to the first generation, experimentally infecting them with *Plasmodium* gametocyte carriers, and assessing the efficacy of type II pyrethroids at both the oocyst and sporozoite stages. This approach will help eliminate biases related to physiological status or age and confirm the trends observed in this study. Nevertheless, the results obtained here remain highly relevant, as they reflect real-world field conditions.

Importantly, these findings align with the recent shift in malaria vector control strategies toward next-generation insecticide-treated nets (ITNs) that combine type II pyrethroids with other insecticides, such as chlorfenapyr (e.g., PermaNet Dual, Interceptor G2) or pyriproxyfen (e.g., Royal Guard). Our results provide robust support for these innovations, underscoring the continued relevance of type II pyrethroids in the ongoing fight against malaria.

### The L119F-*GSTe2* mutation enhances the vectorial competence of wild *Anopheles funestus* (F_0_), whereas the G454A-*CYP9K1* mutation exhibits the opposite effect

Data from both collection sites reveal a strong positive association between the L119F*-GSTe2* mutation and the ability of field-collected mosquitoes to harbor *Plasmodium* and support its development to the infective stage. Similar findings have been reported in Cameroon, where this mutation has been linked to either an increase in the entomological inoculation rate (EIR) [7], or heightened mosquito permissiveness to *Plasmodium* infection [34, 35]. These observations can be explained through several mechanisms. First, mosquito defense against *Plasmodium* involves multiple biological processes, including the production of reactive oxygen species (ROS), a toxic by-products of cellular metabolism that generate oxidative stress and play a critical role in limiting parasite invasion [71]. During *Plasmodium* development, elevated ROS levels are synthesized in the mosquito hemolymph, which typically restricts parasite survival [70, 72]. However, in response to high ROS production, mosquitoes produce potent antioxidant enzymes, such as glutathione S-transferases (GSTs) [73], to detoxify ROS and ensure their own survival. Paradoxically, this detoxification creates an environment more favorable for *Plasmodium* development [74]. Second, the resistance conferred by the *L119F-GSTe2* mutation, as demonstrated in this study, may further explain this phenomenon. *An. funestus s.s.* mosquitoes carrying the 119F-GSTe2 allele are likely better equipped to withstand insecticide exposure in the field, increasing their chances of surviving long enough to transmit *Plasmodium* parasites. Third, resource-based trade-offs may compromise mosquito immune competency. The overproduction of detoxifying enzymes like GSTs could deplete the mosquito’s resource pool, limiting its ability to mount a robust immune response and inadvertently favoring parasite development [74].

In contrast to L119F*-GSTe2*, the G454A**-***CYP9K1* mutation showed the opposite trend: mosquitoes carrying the susceptible allele (SS and RS) were more infected than homozygous resistant (RR) individuals. Unlike GSTs, increased cytochrome P450 monooxygenase activity can lead to an excess of toxic ROS [71, 74]. Combined with the high ROS production resulting from hemoglobin digestion after feeding on infected blood [71], this may create an inhospitable environment for *Plasmodium* development in resistant mosquitoes compared to susceptible ones.

### Pyrethroid resistance escalation potentially induces an increased transmission of *Plasmodium* spp. by *Anopheles funestus* s.l.

The results obtained showed that sporozoite infection rates increased with the level of permethrin resistance, highlighting the fact that the resistance escalation or super-resistance could be associated with a greater risk of *Plasmodium* spp. transmission. Given the fact that the L119F-*GSTe2* resistance marker was simultaneously associated with permethrin resistance escalation and an increasing risk of *Plasmodium* transmission, two main explanations could therefore be considered. One being that the higher the level of permethrin resistance in these mosquitoes, the longer they live and reach the extrinsic incubation period of parasites. The second is that the high level of *GSTe2* expression in these mosquitoes helps them to better metabolize the ROS from both insecticide exposure in the wild and pathogen replication, creating an environment more conducive to the development of *Plasmodium* parasites. This is confirmed by the fact that when exposed to deltamethrin at the dose of insecticide where the L119F-*GSTe2* mutation was found to be significantly associated with resistance (at 1x), the sporozoite infection rate was higher. However, this rate subsequently decreased significantly at insecticide concentrations (5 × and 10 × DD) at which this marker was not associated with resistance or mosquito survival.

## Conclusion

This study underscores the critical role of *An. funestus* as a predominant malaria vector in Cameroon, even alongside the more recognized *An. gambiae*. This wild *An. funestus* (F_0_) population demonstrated alarming levels of pyrethroid resistance, exacerbating the risk of *Plasmodium* transmission. This threat is compounded by the L119F-*GSTe2* mutation, which not only bolsters mosquito survival under insecticide pressure but also amplifies the risk of *Plasmodium* transmission, posing a dual challenge to malaria elimination efforts. On a promising note, the study also validates the efficacy of type II pyrethroids (deltamethrin and alphacypermethrin) against pyrethroid-resistant, *Plasmodium*-infected *An. funestus* populations. This supports the strategic shift toward next-generation insecticide-treated nets, which combine type II pyrethroids with complementary insecticides like chlorfenapyr proven effective against resistant mosquitoes (e.g. Interceptor G2, Permanet Dual). Altogether, these findings highlight the critical importance of resistance management strategies, including insecticide rotation, to curb the escalating threat of pyrethroid resistance that promote malaria transmission.

## Supplementary Information

Supplementary material 1. Figure S1: Impact of the L119F-*GSTe2* mutation on alphacypermethrin (type II pyrethroid) resistance in *Anopheles funestus* s.s. from Mibellon, Panels (**a, b**) show associations between L119F-*GSTe2* genotypes (L/L119-SS, L119F-RS, 119F/F-RR) and survival following exposure to the 1 × diagnostic dose (DD) of alphacypermethrin at the genotypic level, while panels (**c, d**) present the corresponding allelic-level analysis (L119 vs 119F). Panels (**e, f**) and (**g, h**) show genotypic and allelic associations following exposure to 5 × DD, respectively, and panels (**i, j**) and (**k, l**) show genotypic and allelic associations following exposure to 10 × DD. Associations between genotype/allele and survival were assessed using Pearson’s Chi-squared and odds ratios (ORs) with 95% confidence intervals (CI). Statistical significance was set at *P* < 0.05. Figure S2: Impact of the L119F-*GSTe2* mutation on type II pyrethroid (deltamethrin and alphacypermethrin) resistance in *Anopheles funestus* s.s. from Elende. Panels (**a, b**) show associations between L119F-*GSTe2* genotypes (L/L119-SS, L119F-RS, 119F/F-RR) and survival following exposure to the 1 × diagnostic dose (DD) of deltamethrin at the genotypic level, while panels (**c, d**) present the corresponding allelic-level analysis (L119 vs 119F). Panels (**e, f**) and (**g, h**) show genotypic and allelic associations following exposure to 1 × DD of alphacypermethrin. Associations between genotype/allele and survival were assessed using Pearson’s Chi-squared and odds ratios (ORs) with 95% confidence intervals (CI). Statistical significance was set at *P* < 0.05.

## Data Availability

All data generated or analysed during this study are included in this published article [and its supplementary information files].

## Abbreviations

PCR: Polymerase chain reaction
AS-PCR: Allele specific PCR
SIR: Sporozoite infection rate
LLINs: Long lasting insecticide nets
IR: Insecticide resistance
GST: Glutathione S-transferase
*GSTe2*: Glutathione S-transferase epsilon 2
CYP450: Cytochrome P450
*CYP9K1*: Cytochrome P450 9K1
DD: Diagnostic dose
5 × DD: 5 Times the diagnostic dose
10 × DD: 10 Times the diagnostic dose
DEM: Diethyl maleate
PBO: Piperonyl butoxide
F_0_: Generation zero (wild type)
RR: Homozygous resistant
RS: Heterozygous
SS: Homozygous susceptible
