# Supplementary material for: Escalating pyrethroid resistance in Anopheles funestus increases Plasmodium transmission
Source: Malar J. 2026 Aug 13;25:408. doi: 10.1186/s12936-026-06094-6 (PMC13753160; doi:10.1186/s12936-026-06094-6)
Supplement: Supplementary file 1 [file 12936_2026_6094_MOESM1_ESM.docx]

**Supplementary file**

**Figure S1**: Impact of the L119F-*GSTe2* mutation on alphacypermethrin (type II pyrethroid) resistance in *Anopheles funestus* s.s. from Mibellon.

Panels (**a, b**) show associations between L119F-*GSTe2* genotypes (L/L119-SS, L119F-RS, 119F/F-RR) and survival following exposure to the 1× diagnostic dose (DD) of alphacypermethrin at the genotypic level, while panels (**c, d**) present the corresponding allelic-level analysis (L119 vs 119F). Panels (**e, f**) and (**g, h**) show genotypic and allelic associations following exposure to 5× DD, respectively, and panels (**i, j**) and (**k, l**) show genotypic and allelic associations following exposure to 10× DD. Associations between genotype/allele and survival were assessed using Pearson’s Chi-squared and odds ratios (ORs) with 95% confidence intervals (CI). Statistical significance was set at *P* < 0.05.

**Figure S2:** Impact of the L119F-*GSTe2* mutation on type II pyrethroid (deltamethrin and alphacypermethrin) resistance in *Anopheles funestus* s.s. from Elende.

Panels (**a, b**) show associations between L119F-*GSTe2* genotypes (L/L119-SS, L119F-RS, 119F/F-RR) and survival following exposure to the 1× diagnostic dose (DD) of deltamethrin at the genotypic level, while panels (**c, d**) present the corresponding allelic-level analysis (L119 vs 119F). Panels (**e, f**) and (**g, h**) show genotypic and allelic associations following exposure to 1× DD of alphacypermethrin. Associations between genotype/allele and survival were assessed using Pearson’s Chi-squared and odds ratios (ORs) with 95% confidence intervals (CI). Statistical significance was set at *P* < 0.05.
